# Targeting the MYC Oncogene in Burkitt Lymphoma through HSP90 Inhibition

**DOI:** 10.3390/cancers10110448

**Published:** 2018-11-16

**Authors:** Candace J. Poole, Wenli Zheng, Haesung Lee, Danielle Young, Atul Lodh, Ahmed Chadli, Jan van Riggelen

**Affiliations:** 1 Department of Biochemistry and Molecular Biology, Augusta University, 1410 Laney-Walker Blvd., Augusta, GA 30912, USA; capoole@augusta.edu (C.J.P.); zhengwenli@nanodigmbio.com (W.Z.); halee@gwinnettmedicalcenter.org (H.L.); danielle.young@jax.ufl.edu (D.Y.); alodh@augusta.edu (A.L.); 2 Georgia Cancer Center, Augusta University, 1410 Laney-Walker Blvd., Augusta, GA 30912, USA; achadli@augusta.edu

**Keywords:** MYC, HSP90, 17-DMAG, 17-AAG, Burkitt lymphoma

## Abstract

Overexpression of the MYC oncogene is a key feature of many human malignancies including Burkitt lymphoma. While MYC is widely regarded to be a promising therapeutic target, a clinically effective MYC inhibitor is still elusive. Here, we report an alternative strategy, targeting MYC indirectly through inhibition of the HSP90 machinery. We found that inhibition of HSP90 function reduces MYC expression in human Burkitt lymphoma through suppression of MYC transcription and destabilization of MYC protein, thereby diminishing the proliferation of tumor cells. Consistently, treatment of Burkitt lymphoma cell lines with HSP90 inhibitors (17-AAG or 17-DMAG) was accompanied by downregulation of canonical MYC target genes. Combination treatment with 17-DMAG and the proteasome inhibitor, MG-132, led to accumulation of MYC protein, indicating that upon HSP90 inhibition, MYC is degraded by the proteasome. Using co-immunoprecipitation, we furthermore demonstrated a direct interaction between MYC and HSP90, indicating that MYC is an HSP90 client protein in Burkitt lymphoma. Together, we report here the use of HSP90 inhibitors as an alternative approach to target the MYC oncogene and its network in Burkitt lymphoma.

## 1. Introduction

The c-MYC oncogene (herein referred to as MYC) is an important driver of neoplastic transformation and is implicated in the pathogenesis of a wide variety of human cancers (reviewed in [1,2]). The constitutive expression of MYC, caused by genomic rearrangement of the gene, is a frequent abnormality and major oncogenic driver in many aggressive hematopoietic malignancies including Burkitt lymphoma [3,4]. The neoplastic properties of MYC depend on its ability to deregulate a large number of genes, resulting in autonomous cellular growth and proliferation, blocked differentiation, genomic destabilization, and increased angiogenesis (reviewed in [1,5,6]). Its wide implications in human cancers and the notion that tumors can be dependent on MYC expression (oncogene addiction) make MYC and its network a highly promising target for therapeutic strategies [7,8,9]. However, despite considerable efforts in this regard, effective pharmacologic MYC inhibitors are still elusive in the clinical setting. Hence, alternative strategies are much needed. As an alternative approach, the targeted inhibition of protein-protein interactions critical for the function or stability of MYC, such as the MYC-HSP90 axis, might be a promising strategy.

The heat shock protein (HSP) machinery is a well-established therapeutic target for cancer treatment. HSP90 and its co-chaperones are required for the proper folding and functioning of regulatory proteins that contribute to neoplastic properties of tumor cells [10]. Inhibitors of HSP90 such as geldanamycin and its derivatives, as well as purine-scaffold byproducts, are effective in treating different tumor types including, but not limited to: melanoma, multiple myeloma, breast and prostate cancers [11]. These drugs competitively bind the N-terminal ATP site of HSP90, causing proteasomal degradation of HSP90-dependent client proteins [10,11,12]. Interestingly, MYC has been reported to be a client of HSP90 in mantle cell lymphoma [13], and inhibition of HSP90 function has been shown to cause destabilization of MYC and NMYC proteins in neuroblastoma, which was associated with decreased tumor cell proliferation, cell cycle arrest, increased apoptosis, and upregulation of tumor suppressors, such as p53 [14]. Furthermore, it has been shown that HSP90 protein expression is elevated in Burkitt lymphoma and may provide as a target for therapeutic intervention [15]. Based on these reports, we speculated that the MYC-HSP90 axis might be critical for tumor maintenance in Burkitt lymphoma, potentially providing a new therapeutic strategy.

Here, we report that in Burkitt lymphoma the MYC oncogene can be targeted indirectly through inhibition of the HSP90 machinery, leading to both downregulation of MYC transcription and decreasing MYC protein stability, resulting in diminished tumor cell proliferation through cell cycle arrest, necrosis and apoptosis.

## 2. Results

### 2.1. 17-AAG Treatment Downregulates MYC Expression in Burkitt Lymphoma

**Figure 1:**
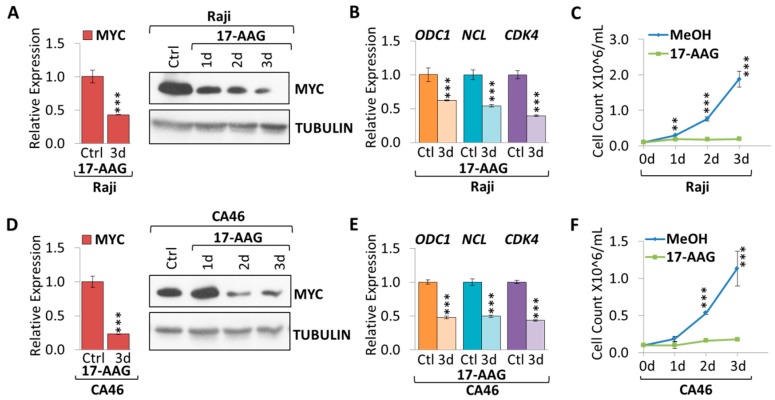
17-AAG treatment suppresses MYC in Burkitt lymphoma. RT-qPCR and Western Blot (WB) of MYC expression upon 4 µM 17-AAG treatment over the course of three days in (**A**) Raji and (**D**) CA46 cell lines. RT-qPCR of canonical MYC target genes: *ODC1*, *NCL*, and *CDK4* in (**B**) Raji and (**E**) CA46 cell lines upon three days treatment of 4 µM 17-AAG or MeOH. Growth curve of cells treated with MeOH or 4 µM 17-AAG over the course of three days in (**C**) Raji and (**F**) CA46 cell lines. RT-qPCR was normalized to *RPL13a*; Tubulin is used as a WB loading control. Error bars represent mean ± SEM; n = 3; two-tailed Student’s *t*-test: * *p* < 0.05; ** *p* < 0.01; *** *p* < 0.001.

In order to determine the effect HSP90 function has on MYC expression in Burkitt lymphoma, we disrupted HSP90 function by using the commercially available HSP90 inhibitor, 17-N-allylamino-17-demethoxygeldanamycin (17-AAG), a derivative of geldanamycin [16]. First, we tested the effect of various concentrations of 17-AAG, ranging from 0.5–6 µM, on Raji cells, by monitoring cell growth (Supplementary Figure S1); the growth curves indicate a direct correlation to the dose of 17-AAG. Based on the pilot experiment, we chose a 4 µM 17-AAG dose to treat Burkitt lymphoma cell lines (Raji, CA46, and Daudi) over the course of three days, comparing them to methanol as a control (MeOH) (Figure 1). We found a significant decrease in MYC mRNA (2.3-fold for Raji, and 4.3-fold for CA46) and protein for all cell lines treated with 17-AAG compared to MeOH (Figure 1A,D and Supplementary Figure S2). Suppression of MYC caused downregulation of canonical MYC target genes, *CDK4* (2.5-fold for Raji, and 2.3-fold for CA46), *NCL* (1.8-fold for Raji, and 2.0-fold for CA46), and *ODC1* (1.6-fold for Raji, and 2.1-fold for CA46) (Figure 1B,E). 17-AAG treatment significantly reduced tumor cell proliferation compared to MeOH over the course of three days in all cell lines (Figure 1C,F and Supplementary Figure S2).

**Figure 2:**
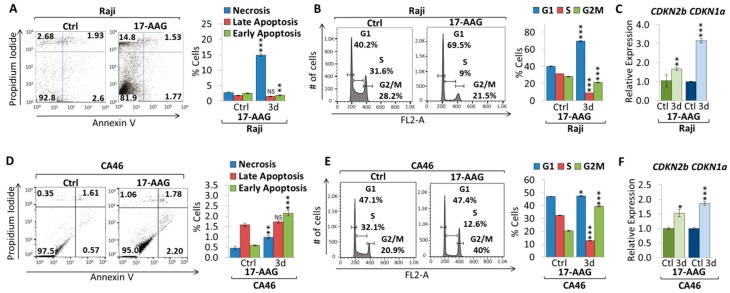
17-AAG treatment causes apoptosis and cell cycle arrest in Burkitt lymphoma. Flow cytometric analysis of apoptosis using AnnexinV/PI staining. Flow cytometry profile of AnnexinV staining (X axis) and PI (Y axis) is shown for (**A**) Raji and (**D**) CA46 cell lines upon three days treatment with 4 µM 17-AAG. The lower right quadrant indicates the percentage of early apoptotic cells in each condition; the upper right quadrant indicates the percentage of late apoptotic cells; the upper left quadrant indicates percentage of necrotic cells; and the left lower quadrant indicates percentage of live/non-apoptotic cells. Apoptotic cells (Annexin V-positive cells) are displayed as the percentage of gated cells. Flow cytometric cell cycle analysis using propidium iodide (PI) staining in (**B**) Raji and (**E**) CA46 cell lines upon three days treatment with 4 µM 17-AAG. Cell cycle distribution (G1, S and G2/M) are displayed in percent. RT-qPCR of *CDKN2b* and *CDKN1a* upon three-day treatment of 4 µM 17-AAG or MeOH in (**C**) Raji and (**F**) CA46 cell lines. RT-qPCR was normalized to *RPL13a*. Error bars represent mean ± SEM; n = 3; two-tailed Student’s *t*-test: NS = not significant; * *p* < 0.05; ** *p* < 0.01; *** *p* < 0.001.

To further elucidate the mechanism underlying 17-AAG treatment of Burkitt lymphoma cell lines, apoptosis and cell cycle analyses were carried out (Figure 2). AnnexinV/ PI staining indicates increases in the percentage of cells undergoing early apoptosis (0.6% to 2.2% in CA46) and late apoptosis (1.6% to 1.7% in CA46). This result is consistent with the effect of 17-AAG on Daudi cells (see Supplementary Figure S2). In contrast, Raji cells decreased the percentage of cells in early apoptosis (2.5% to 1.8%) and late apoptosis (1.7% to 1.4%), although not significantly. In parallel, we observed an increase in necrotic cells in all cell lines (2.7% to 14.8% for Raji, and 0.5% to 1.0% for CA46) (Figure 2A,D and Supplementary Figure S2). Flow cytometric cell cycle analysis using propidium iodide (PI) staining of Raji and Daudi cell lines upon three days treatment with 4 µM 17-AAG indicates a cell cycle arrest in G1 phase, while S phase dramatically decreased (Figure 2B and Supplementary Figure S2). In contrast, CA46 cells indicate a cell cycle arrest in G2 phase instead of G1, while S phase decreased upon three days treatment with 4 µM 17-AAG (Figure 2E). We detected an increase in mRNA expression for the cell cycle-dependent kinase inhibitor *CDKN2b* in all cells lines (1.53-fold in CA46, and 1.66-fold in Raji); Furthermore, *CDKN1a* mRNA expression increased in CA46 and Raji cells (1.87-fold and 3.15-fold, respectively), but this was not observed in Daudi cells (Figure 2C,F and Supplementary Figure S2). Together, our results show that 17-AAG decreased tumor cell proliferation and reduced MYC mRNA and protein expression, subsequently causing both cell cycle arrest and apoptosis in Burkitt lymphoma cell lines.

### 2.2. 17-DMAG Treatment Downregulates MYC Expression in Burkitt Lymphoma

Since 17-AAG was effective in suppressing MYC mRNA and protein expression while inhibiting tumor cell growth, we validated our results using another geldanamycin derivative, 17-dimethylaminoethylamino-17-demethoxy-geldanamycin (17-DMAG). 17-DMAG is reported to have better solubility than 17-AAG and is less toxic to normal cells compared to tumor cells [17]. We tested the effect of 0.1–2 µM 17-DMAG on Raji cells, by monitoring cell growth (Supplementary Figure S1). The growth curves indicate a direct correlation to the dose of 17-DMAG. Previous studies testing the viability of normal B cells in response to increasing doses of 17-DMAG for 24 and 48 h indicate that 1 µM 17-DMAG is not toxic, so for further experiments we used a 1 µM dose [18].

**Figure 3:**
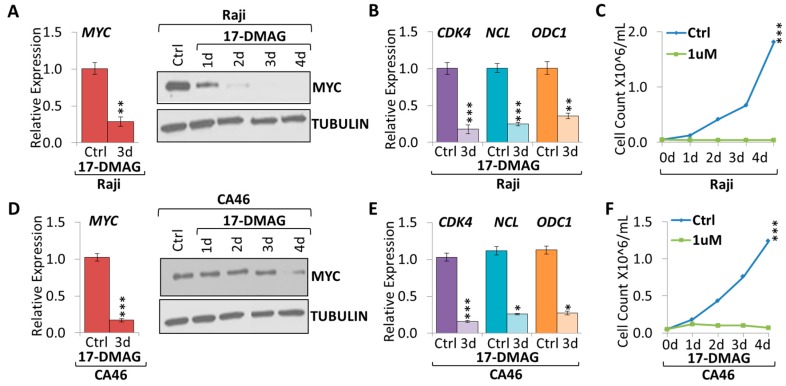
17-DMAG treatment downregulates MYC expression in Burkitt lymphoma. RT-qPCR and WB of MYC expression upon 1 µM 17-DMAG treatment over the course of four days in (**A**) Raji and (**D**) CA46 cell lines. RT-qPCR of canonical MYC target genes: *CDK4*, *NCL*, and *ODC1* in (**B**) Raji and (**E**) CA46 cell lines upon three days treatment of 1 µM 17-DMAG compared to untreated control. Growth curve of cells treated with 1 µM 17-DMAG over the course of four days in (**C**) Raji and (**F**) CA46 cell lines. RT-qPCR was normalized to *RPL13a*; Tubulin is used as a WB loading control. Error bars represent mean ± SEM; n = 3; two-tailed Student’s *t*-test: * *p* < 0.05; ** *p* < 0.01; *** *p* < 0.001.

To investigate if 17-DMAG is capable of suppressing MYC, we treated Burkitt lymphoma cell lines with 1 µM 17-DMAG over the course of four days (Figure 3). We found a significant decrease in MYC mRNA and protein expression in Burkitt lymphoma cell lines treated with 17-DMAG compared to wildtype untreated control (Figure 3A,D). MYC mRNA was 3.5-fold and 4.9-fold lower upon three-day treatment with 1 µM 17-DMAG in both Raji and CA46 cell lines, respectively. 17-DMAG treatment caused a significant downregulation of canonical MYC target genes, *CDK4*, *NCL*, and *ODC1* (Figure 3B,E). *CDK4* mRNA was 5.6-fold and 5.2-fold lower, *NCL* mRNA was 4.1-fold and 3.3-fold lower, and *ODC1* mRNA was 2.8-fold and 3.2-fold lower upon three days treatment with 1 µM 17-DMAG in both Raji and CA46 cell lines, respectively. Accompanying MYC suppression we found significantly reduced tumor cell proliferation (Figure 3C,F).

**Figure 4:**
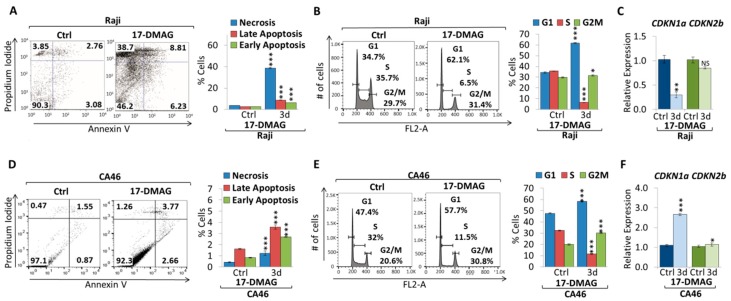
17-DMAG treatment causes apoptosis and cell cycle arrest in Burkitt lymphoma. Flow cytometric analysis of apoptosis using AnnexinV/PI staining. Flow cytometry profile of AnnexinV staining (X axis) and PI (Y axis) is shown for (**A**) Raji and (**D**) CA46 cell lines upon three days treatment with 1 µM 17-DMAG compared to untreated control. The lower right quadrant indicates the percentage of early apoptotic cells in each condition; the upper right quadrant indicates the percentage of late apoptotic cells; the upper left quadrant indicates percentage of necrotic cells; and the left lower quadrant indicates percentage of live/non-apoptotic cells. Apoptotic cells (Annexin V-positive cells) are displayed as the percentage of gated cells. Flow cytometric cell cycle analysis using propidium iodide (PI) staining in (**B**) Raji and (**E**) CA46 cell lines upon three days treatment with 1 µM 17-DMAG compared to untreated control. Cell cycle distribution (G1, S and G2/M) are displayed in percent. RT-qPCR of *CDKN1a* and *CDKN2b* upon three days treatment of 1 µM 17-DMAG compared to untreated control in (**C**) Raji and (**F**) CA46 cell lines. RT-qPCR was normalized to *RPL13a.* Error bars represent mean ± SEM; n = 3; two-tailed Student’s *t*-test: NS = not significant; * *p* < 0.05; ** *p* < 0.01; *** *p* < 0.001.

To further elucidate the underlying mechanism of 17-DMAG treatment of Burkitt lymphoma cell lines, we performed apoptosis and cell cycle analyses (Figure 4). AnnexinV/ PI staining indicates in both cell lines increasing early apoptosis (2.5% to 6.2% in Raji cells, and 0.8% to 2.2% in CA46 cells) and late apoptosis (2.5% to 8.7% in Raji cells, and 1.6% to 1.7% in CA46 cells), while we observed an increase in necrotic cells (3.6% to 38.8% for Raji cells, and 0.4% to 1.0% for CA46 cells) (Figure 4A,D). Flow cytometric cell cycle analysis using propidium iodide (PI) staining in Raji and CA46 cell lines upon three days treatment with 1 µM 17-DMAG indicates cell cycle arrest, with an increase in the percentage of cells in both G1 and G2/M phase, while the percentage of cells in S phase significantly decreased (Figure 4B,E). In parallel, we observed an increase in cell cycle-dependent kinase inhibitors, *CDKN1a* (p21cip1) and *CDKN2b* (p15ink4b), expression only in CA46 cells (3.1-fold and 1.4-fold, respectively), while Raji cells exhibited decreased expression levels (3.4-fold and 1.2-fold, respectively) (Figure 4C,F). Together, our results show that 17-DMAG decreased tumor cell proliferation and reduced MYC mRNA and protein expression, ultimately causing cell cycle arrest, necrosis and apoptosis in Burkitt lymphoma cell lines.

### 2.3. 17-DMAG Treatment Decreases Active, While Increasing Inactive Chromatin Marks at the Translocated MYC Locus in Burkitt Lymphoma

To elucidate the mechanism underlying MYC suppression, we further evaluated how translocated *MYC* is transcriptionally suppressed following 17-DMAG treatment. Burkitt lymphomas are characterized by constitutive MYC expression caused by one of three chromosomal translocations, placing *MYC* under control of the immunoglobulin heavy chain (IgH) enhancer [3,4]. The most common variant is t(8;14)(q24;q32), which accounts for about 85% of all cases. Interestingly, HSP90 has recently been implicated in transcriptional regulation through epigenetic processes. In particular, a direct interaction has been shown between HSP90 and Trithorax group (TrxG) proteins which induce transcription through tri-methylation of K4 on histone H3 (H3K4me3) [19].

**Figure 5:**
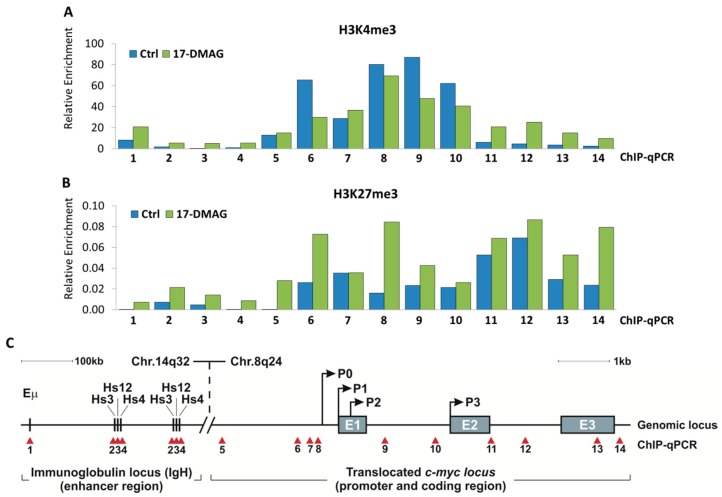
17-DMAG treatment alters chromatin status at the translocated MYC locus in Burkitt lymphoma. ChIP-qPCR for (**A**) H3K4me3 modifications and (**B**) H3K27me3 modifications at the MYC locus upon 1 µM 17-DMAG treatment for three days. (**C**) Visual schematic of ChIP-qPCR tiling amplicons in the immunoglobulin enhancers: *Eµ*, *Hs3*, *Hs12*, and *Hs4* (#1–4) of chromosome 14, and in the translocated *MYC* promoter (#5–10) and the *MYC* locus (#11–14) of chromosome 8. Red arrows indicate placement of amplicon. MYC promoters are referred to as “P”, and Exons as “E”. ChIP-qPCR was normalized to Input DNA. Each bar is representative for average mean of duplicate samples.

To investigate the consequence of 17-DMAG treatment on the transcriptional suppression of *MYC*, we used chromatin immunoprecipitation (ChIP)-qPCR to map histone modifications at the translocated *MYC* locus in Burkitt lymphoma (Figure 5). We treated Burkitt lymphoma cell line (Raji) with 1 µM 17-DMAG for three days and found decreased enrichment of active chromatin marks, H3K4me3, compared to untreated control at the translocated *MYC* locus region (Figure 5A). In parallel, we found increased enrichment of inactive chromatin marks, H3K27me3, compared to untreated control at the *MYC* locus (Figure 5B). ChIP-qPCR tiling amplicons for translocated *MYC* in Burkitt lymphoma were mapped over the immunoglobulin enhancers *Eµ*, *Hs3*, *Hs12*, and *Hs4* on chromosome 14 (Amplicons 1–4), as well as, the *MYC* locus on chromosome 8 (Amplicons 5–14) (Figure 5C). Together, our data support a model in which HSP90 inhibition suppresses *MYC* transcription through a chromatin switch, from active to inactive histone modifications, at the translocated *MYC* locus.

### 2.4. MYC Is a HSP90 Client Protein in Burkitt Lymphoma

**Figure 6:**
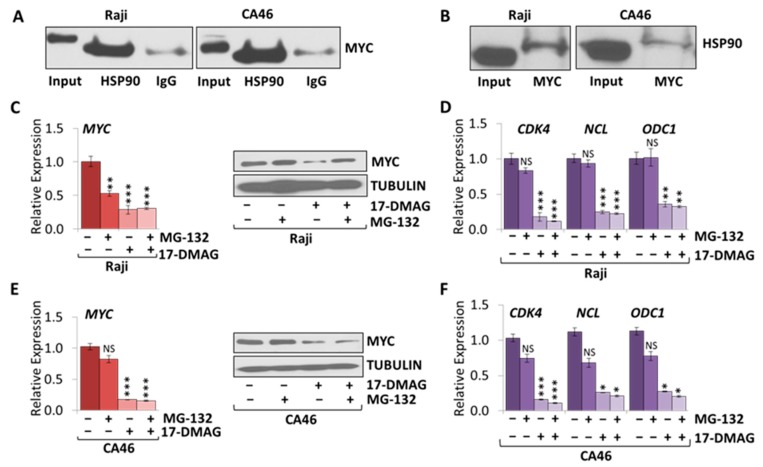
17-DMAG treatment causes proteasomal degradation of MYC protein in Burkitt Lymphoma. Co-IP was performed in Burkitt lymphoma cell lines, Raji and CA46. (**A**) HSP90 was immunoprecipitated and blotted for MYC expression. (**B**) MYC was immunoprecipitated and blotted for HSP90 expression. RT-qPCR and WB of MYC expression upon 1 µM 17-DMAG and 10 µM MG-132 combination treatment over four hours in (**C**) Raji and (**E**) CA46 cells. RT-qPCR of canonical MYC target genes, *CDK4*, *NCL*, and *ODC1* upon three days treatment with 1 µM 17-DMAG in (**D**) Raji and (**F**) CA46 cells. RT-qPCR was normalized to *RPL13a*; Tubulin was used as a loading control for WB. Error bars represent mean ± SEM; n = 3; two-tailed Student’s *t*-test: NS = not significant; * *p* < 0.05; ** *p* < 0.01; *** *p* < 0.001.

MYC has first been reported to be a client protein of HSP90 in mouse fibroblasts [20]. More recently, the MYC-HSP90 axis has been shown to be important in a variety of cancers, including but not limited to, breast cancer stem cells and mantle cell lymphoma [13,21]. To determine if HSP90 physically interacts with MYC in Burkitt lymphoma, we performed co-immunoprecipitation (Co-IP). HSP90 protein was immunoprecipitated with a monoclonal antibody directed against HSP90, while rabbit IgG was used as a negative control. We then immunoblotted for MYC expression, confirming an interaction between MYC and HSP90 in both Raji and CA46 cell lines (Figure 6A). Using the opposite approach to confirm these results, we immunoprecipitated MYC protein using a monoclonal antibody directed against MYC, and followed by immunoblotting for HSP90 (Figure 6B). Together, our results show that in Burkitt lymphoma, MYC and HSP90 exist in a complex with each other, suggesting that HSP90 contributes to MYC protein stability.

To further support the notion that HSP90 stabilizes MYC in Burkitt lymphoma, we combined HSP90 inhibition with inhibition of the proteasome. We treated Burkitt lymphoma cell lines with 1 µM 17-DMAG in combination with 10 µM of the cell permeable proteasome inhibitor, MG-132, for four hours. We found a significant decrease in MYC mRNA and protein expression in all cell lines treated with 17-DMAG versus untreated control. Combination treatment of 17-DMAG and MG-132 did not further reduce mRNA expression levels in cells but protein degradation was attenuated. Our results indicate that 17-DMAG downregulates MYC in a proteasomal degradation-dependent manner in Raji and CA46 cells (Figure 6C,E). Furthermore, 17-DMAG treatment caused downregulation of canonical MYC target genes, *CDK4*, *NCL*, and *ODC1* in the presence of, and without 10 µM MG-132, in Raji and CA46 cells (Figure 6D,F). The finding that, in the presence of 17-DMAG, MG-132 did not rescue MYC target gene expression, suggests that accumulated, but ubiquitinated MYC is not transcriptionally functional.

HSP90 inhibitors have been shown to have clinical activity in other hematologic malignancies including acute lymphoblastic leukemia (ALL), and acute myeloid leukemia (AML) [22,23]. Since ALL, AML (in some cases) and chronic myeloid leukemia (CML) (through BCR-ABL) are also MYC-positive, we tested whether HSP90 inhibition would downregulate MYC in the human CML cell line, K562 (Supplementary Figure S3). Indeed, we found that both MYC mRNA and protein levels decrease upon 17-AAG treatment, while MYC protein was restored by additionally blocking the proteasome with MG-132. Together, this indicates that HSP90 inhibition affects transcription and protein stability of MYC similar to Burkitt lymphoma, even if the *c-myc* gene is not translocated. Our results show that in the absence of HSP90 function, MYC is destabilized and degraded by the proteasome pathway. Together, we conclude that MYC is a client protein of HSP90 in Burkitt lymphoma.

## 3. Discussion

Here, we report that in Burkitt lymphoma the MYC oncogene is a client of HSP90 and can be targeted indirectly through inhibition of the HSP90 machinery. Deregulated expression of MYC is recognized as the Achilles heel of many cancer types and hence is widely regarded to be a promising therapeutic target [8,9]; however, effective pharmacological MYC inhibitors are elusive, even leading to the conclusion that MYC might be ‘undruggable’ [24,25]. However, targeting protein-protein interactions between MYC and its co-regulatory factors, such as the MYC-HSP90 axis, might be a promising alternative strategy. Here, we provide evidence that in Burkitt lymphoma the MYC oncogene is a client protein of HSP90, and that MYC expression can be targeted by inhibiting HSP90 function with readily available pharmacologic inhibitors.

Burkitt lymphomas are a paradigm of MYC dysregulation, overexpressing MYC due to chromosomal translocation placing the gene under transcriptional control of the immunoglobulin heavy chain enhancer [3,4]. Since MYC has been reported to be a client of HSP90 in mantle cell lymphoma [13], and HSP90 inhibition caused protein destabilization of MYC and NMYC in neuroblastoma [14], we speculated that the MYC-HSP90 axis might also be critical in Burkitt lymphoma. Exploiting this notion, we found that treating human Burkitt lymphoma cell lines (Raji and CA46) with the HSP90 inhibitors, 17-AAG or 17-DMAG, decreased MYC expression levels by reducing both MYC mRNA and protein levels, subsequently diminishing tumor cell proliferation causing cell cycle arrest, and increasing necrosis and apoptosis.

Considering both transcriptional and post-translational mechanisms of action, we found that 17-DMAG treatment caused changes in the transcriptional regulation of the translocated *MYC* gene in Burkitt lymphoma cells, revealing a shift from active to inactive chromatin structure. Using ChIP-qPCR tiling of the immunoglobulin enhancers *Eµ*, *Hs3*, *Hs12*, and *Hs4* on chromosome 14, as well as the translocated *MYC* gene on chromosome 8, we detected a decrease in H3K4me3 enrichment as an active chromatin mark, while H3K27me3 enrichment as an inactive chromatin mark increased. The relatively modest change in enrichment for H3K4me3 and H3K27me3 might be due to the only short time 17-DMAG treatment. Alternatively, one could speculate about the *c-myc* promoter exhibiting an epigenetic memory of HSP90 inhibition (reviewed in [26]). Our results relate to the notion that HSP90 has recently been implicated in transcriptional regulation through epigenetic processes. In particular, a direct interaction has been shown between HSP90 and Trithorax group (TrxG) proteins which induce transcription through H3K4me3 [19]. Together, this provides a possible explanation for how HSP90 inhibition causes MYC mRNA levels to decrease through a transcriptional mechanism, involving an epigenetic switch. Further experiments would be necessary to answer whether HSP90 together with TrxG proteins directly bind to the immunoglobulin enhancers or the *MYC* locus. It also remains to be seen, whether this mechanism would apply to non-translocated *MYC* in other cancer types.

In parallel to the switch in MYC transcription, we found that in Burkitt lymphoma cells, MYC is indeed a client protein of HSP90 and HSP90 inhibition affects MYC expression through protein destabilization. The combination of 17-DMAG treatment with the proteasome inhibitor MG-132, however, restored MYC protein levels, indicating that proteasomal degradation of MYC is due to protein destabilization through HSP90 inhibition. Furthermore, Co-IP analysis indicates that in Burkitt lymphoma MYC protein exists in complex with HSP90. These findings are consistent with reports that MYC is a client of HSP90 in mantle cell lymphoma [13], and that inhibition of HSP90 function causes destabilization of MYC and NMYC proteins in neuroblastoma [14]. Together, our data show that 17-DMAG destabilizes MYC protein causing proteasomal degradation as well as upstream epigenetic regulation at the translocated *MYC* locus.

HSP90 inhibitors have clinical activity in acute lymphoblastic leukemia (ALL), and acute myeloid leukemia (AML) as well [22,23]. Our results indicate that HSP90 inhibition affects transcription and protein stability of MYC similar to Burkitt lymphoma, even if the c-myc gene is not translocated like in CML. MYC inactivation can cause sustained tumor regression in a variety of cancer types, eliciting oncogene addiction by triggering cellular senescence and apoptosis [7,27,28,29]. We found that suppression of MYC in Burkitt lymphoma upon HSP90 inhibition was associated with an increase in G0/G1 cell cycle arrest, and an increase in necrotic and apoptotic cells. This is consistent with the notion that constitutively expressed MYC is a major oncogenic driver in Burkitt lymphoma, being required for proliferation and survival. Among canonical MYC target genes, many have functions critical for cell cycle progression such as *CDK4*, *CDK6* and *Cyclin D1*, while MYC repressed genes such as *CDKN2B* and *CDKN1A* are negative regulators of the cell cycle [30]. However, MYC is not the only HSP90 client protein in Burkitt lymphoma, and so it is possible that the mechanism of action is not exclusively through MYC, but involves the destabilization of cell cycle regulators such as CDK2 and CDK4 directly due to loss of HSP90 function. Together, we concluded that HSP90 inhibition in Burkitt lymphoma cell lines ultimately caused a drastic reduction in cell proliferation and viability through inducing cell cycle arrest, apoptosis and necrosis. Our findings are consistent with reports that in neuroblastoma and mantle cell lymphoma, where downregulation of MYC upon HSP90 inhibition was associated with loss of tumor cell proliferation, cell cycle arrest, increased apoptosis, and upregulation of tumor suppressors, such as p53 [14,31]. Intriguingly, HSP90 inhibition has been reported to have opposing effects on wild-type and mutant p53 [32]. At the same time, the majority of Burkitt lymphomas (including Raji cells) are known to express a mutant p53 [33,34,35]. Future experiments will need to address the role of p53 during HSP90 inhibition in Burkitt lymphoma. Another group treating Burkitt lymphoma cells with the HSP90 inhibitor, PU176, also reported reduced tumor cell proliferation; however, they suggested the PI3K/AKT/mTOR pathway (but not MYC) as the main mechanism of action [15].

In summary, our results show that in Burkitt lymphoma the MYC oncogene is a client protein of HSP90, and that inhibition of HSP90 function with pharmacologic inhibitors caused a switch in MYC transcription as well as MYC protein destabilization. Together, our findings suggest that targeting MYC indirectly by inhibiting the HSP90 machinery, using 17-DMAG as a potential chemotherapeutic drug, may be an alternative therapeutic strategy for Burkitt lymphoma and possibly other MYC-associated cancers.

## 4. Materials and Methods

### 4.1. Cell Culture

Human Burkitt lymphoma cell lines (Daudi, Raji and CA46) were obtained from ATCC. All leukemia/lymphoma cell lines were passaged less than 8 times, maintained in RPMI1640 supplemented with 10% FBS, 1% penicillin/streptomycin, 1% l-glutamine and 50 μM 2-mercaptoethanol, and incubated at 37 °C humidified with 5% CO_2_.

### 4.2. Treatment Conditions

17-DMAG (Cayman Chemical, Ann Arbor, MI, USA) was dissolved in ultra-pure water and given at a 1 µM dose. MG-132 (Fisher Scientific, Hampton, NH, USA) was dissolved in methanol (MeOH) and given at a 10 µM dose. 17-AAG (Cayman Chemical, Ann Arbor, MI, USA) was dissolved in methanol (MeOH) or dimethyl sulfoxide (DMSO) and given at a 4 µM dose. Small molecule inhibitors or solvent control (MeOH/DMSO) was added to cell culture medium for the indicated times. Cell line viability with Trypan Blue was performed in triplicates using the Nexcelom Bioscience Cellometer2000Auto cell counter with Nexcelom Bioscience™ SD100 counting slides (Nexcelom, Lawrence, MA, USA).

### 4.3. RNA Extraction and Analysis of Gene Expression

Total RNA was isolated using the NucleoSpin RNA Kit including DNase-I digest (Machery-Nagel Inc., Düren, Germany) following the manufacturer’s protocol. 0.5 µg RNA was reverse transcribed into cDNA using the iScript cDNA Kit (BioRad, Hercules, CA, USA). Quantitative PCR was performed using SYBR GREEN (BioRad, Hercules, CA, USA) in an ABI StepOne Plus analyzer. The specific forward (F) and reverse (R) primer sequences are listed in Supplementary Table S1.

### 4.4. Cell Extracts and Western Blot Analysis

Total protein extracts were prepared using a lysis buffer (50 mM Tris, 2% SDS, 10% glycerol, 0.74 M beta-mercaptoethanol), sonicated on ice using a Sonifier 250D (Branson Ultrasonics, St. Louis MO, USA), and heated for 5 min at 99 °C. Protein concentrations were determined with the Bio-Rad DC Protein Assay Kit (BioRad, Hercules, CA, USA) using bovine serum albumin as a standard. Protein extracts were separated in 10% SDS-PAGE, electrotransferred to PVDF membranes (Immobilon-P; EMD Millipore, Burlington, MA, USA). Antibodies used are: HSP90 antibody [H90-10] #ab53497, MYC #ab32 (Abcam, Cambridge, UK) [36], #sc764 (Santa Cruz Biotechnology, Dallas, TX, USA) [37]; TUBULIN #ab6046 (Abcam, Cambridge, UK) [38]; Anti-MOUSE HRP #31430 (Thermo Fisher, Waltham, MA, USA) [39]; Anti-RABBIT HRP #31460 (Thermo Fisher, Waltham, MA, USA) [40].

### 4.5. Cell Cycle Analysis Using Propidium Iodide

Cells were fixed in 70% methanol at −20 °C for a minimum of 72 h and stained using a Propidium Iodide (PI) solution containing PBS+0.5% BSA, 50 µg/mL PI (Acros Organics/Thermo Fisher, Waltham, MA, USA), and 200 µg/mL RNaseA (Thermo Fisher, Waltham, MA, USA). Cells were then analyzed immediately on a FACScalibur flow cytometer (Becton Dickinson, Franklin Lakes, NJ, USA). FACS data were analyzed using FlowJo software (Tree Star, Ashland, OR, USA).

### 4.6. Flow Cytometric Analysis of Cell Death

Annexin V-FITC and PI staining was used for the study of cell cycle distribution and apoptosis using the Annexin V-FITC Early Apoptosis Detection Kit (Cell Signaling, Danvers, MA, USA). Briefly, cells were washed in PBS and suspended in 1X AnnexinV binding buffer. AnnexinV-FITC conjugate and propidium iodide were incubated for 10 min on ice and immediately analyzed on a FACScalibur flow cytometer (Becton Dickinson, Franklin Lakes, NJ, USA). FACS data were analyzed using FlowJo software (Tree Star, Ashland, OR, USA).

### 4.7. Chromatin Immunoprecipitation (ChIP) Analysis

ChIP was performed as previously described [41]. Cells were crosslinked with 1% formaldehyde followed by quenching with 0.2 M glycine. After washing with PBS, the cells were resuspended in lysis buffer (10 mM EDTA pH 8.0, 50 mM Tris-HCl pH 8.0, 1% SDS). Chomatin was sheared by sonication for 10 min using a Branson 250 Sonifier, diluted in ChIP Dilution Buffer (0.01% SDS, 1.1% Triton X-100, 1.2 mM EDTA pH 8.0, 16.7 mM Tris-HCl pH 8.0 and 167 mM NaCl) and incubated with 5 µg of specific antibody overnight. The bound material was recovered after two hours incubation with 50 µL Dynabeads/Protein G (Thermo Fisher, Waltham, MA, USA), rotating at 4 °C. The beads were washed, once in Low Salt Buffer (0.1% SDS, 1% Triton X-100, 2 mM EDTA pH 8.0, 20 mM Tris-HCl pH 8.0 and 150 mM NaCl), twice in High Salt Buffer (0.1% SDS, 1% Triton X-100, 2 mM EDTA pH 8.0, 20 mM Tris-HCl pH 8.0 and 500 mM NaCl), twice in LiCl Buffer (0.25 M LiCl, 1% NP-40, 1% Na-Deoxycholate, 1 mM EDTA pH 8.0 and 10 mM Tris-HCl pH 8) and twice in TE buffer. ChIPed material was eluted by two 15 min incubations at room temperature with 250 µL Elution Buffer (1% SDS and 0.1 M NaHCO_3_). Chromatin was reverse-crosslinked by adding 0.2 M NaCl for four hours at 65 °C. After RNase and proteinase K treatment, DNA was extracted using phenol-chloroform. ChIP followed by quantitative PCR was performed using SYBR GREEN (BioRad, Hercules, CA, USA) in an ABI StepOne Plus analyzer. The specific forward (F) and reverse (R) primer sequences are listed in Supplementary Table S2.

### 4.8. Co-Immunoprecipitation

Cells were washed with PBS and lysed in cold immunoprecipitation buffer (150 mM NaCl, 10 mM Tris-HCl (pH 7.4), 1 mM EDTA, 1 mM EGTA (pH 8.0), 1% Triton X-100, 0.5% NP-40, and fresh proteinase inhibitor cocktail) for 30 min at 4 °C. Cells were sonicated with a Branson digital sonifier for 5 s at 20% intensity four times. For immunoprecipitation, 25 μL Protein A Magnetic Beads were added to 200 μL of crude cell extract incubate for 1 h at 4 °C. Beads were separated by magnets, and supernatant moved to a clean 1.5 mL microcentrifuge tube. 1–5 μg of antibody was added to crude cell lysate and incubated for 1 h at 4 °C. 5 μg rabbit anti-mouse IgG antibody was used as a control. 25 μL of Protein A/G Magnetic Beads suspension was added and incubated with agitation for 1 h at 4 °C. Beads were separated by magnet and were washed 2 times with 500 μL of Immunoprecipitation Buffer. The bead pellet was resuspended in 30 μL of 3X SDS Sample Loading Buffer (187.5 mM Tris-HCl (pH 6.8), 6% (*w*/*v*) SDS, 30% glycerol, 150 mM DTT 0.03% (*w*/*v*), bromophenol blue, 2% β-mercaptoethanol). Sample was incubated for 5 min at 70 °C. Magnetic field was applied to sample and supernatant was loaded on SDS-PAGE for analysis.

### 4.9. Statistical Analysis

All experiments were performed on biological replicates unless otherwise specified. Sample size is reported in the respective figure legends. All quantitative PCR were run in triplicates and standard deviation is shown in the figures. Two-tailed Student’s *t*-test was used to calculate *p*-values; statistically significant values are specified in the figure legends.

## 5. Conclusions

We found that inhibition of HSP90 function drastically decreased MYC expression through suppression of MYC transcription and destabilization of MYC protein in human Burkitt lymphoma cell lines. Treatment with HSP90 inhibitors, 17-AAG or 17-DMAG, was accompanied by downregulation of canonical MYC target genes, decreased tumor cell proliferation, cell cycle arrest, and increased apoptosis and necrosis. Combination treatment with 17-DMAG and proteasome inhibitor accumulated MYC protein, indicating that MYC is degraded by the proteasome upon HSP90 inhibition. Furthermore, using co-immunoprecipitation we demonstrated a direct interaction between MYC and HSP90, indicating that MYC is an HSP90 client protein in Burkitt lymphoma. Together, we demonstrate that the MYC oncogene and its network can be targeted indirectly through the use of HSP90 inhibitors in Burkitt lymphoma.

## Supplementary Materials

The following are available online at http://www.mdpi.com/2072-6694/10/11/448/s1, Figure S1: Dose–response curve for 17-AAG and 17-DMAG treatment on Burkitt Lymphoma, Figure S2: 17-AAG treatment downregulates MYC expression and caused decreased tumor cell proliferation, apoptosis, and cell cycle arrest in Daudi cells, Figure S3: 17-AAG treatment causes MYC downregulation and proteasomal degradation of MYC protein in Chronic Myeloid Leukemia, Table S1: RT-qPCR primers, Table S2: ChIP-qPCR tiling primers.
