# Supplementary material for: Targeting the MYC Oncogene in Burkitt Lymphoma through HSP90 Inhibition
Source: Cancers (Basel). 2018 Nov 16;10(11):448. doi: 10.3390/cancers10110448 (PMC6266960; doi:10.3390/cancers10110448)
Supplement: Supplementary file 1 [file cancers-10-00448-s001.pdf]

cancers

Supplementary Materials: Targeting the MYC Oncogene in Burkitt Lymphoma through HSP90 Inhibition

Candace J. Poole, Wenli Zheng, Haesung Lee, Danielle Young, Atul Lodh, Ahmed Chadli and Jan van Riggelen

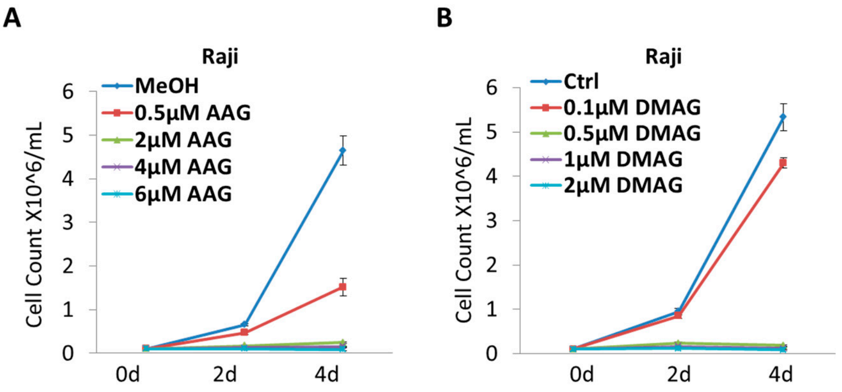

Figure S1. Dose response curve for 17-AAG and 17-DMAG treatment on Burkitt Lymphoma. Dose response growth curve on Raji Burkitt Lymphoma cell line with (**A**) 0.5-6μM 17-AAG compared to Methanol (MeOH) control; and (**B**) 0.1-2μM 17-DMAG compared to untreated control.

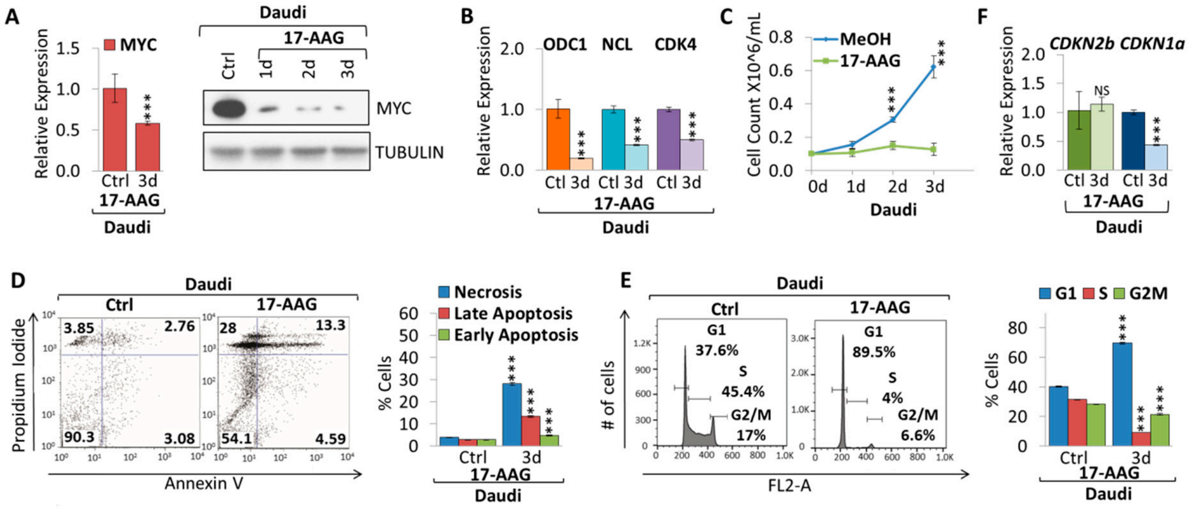

Figure S2. 17-AAG treatment downregulates MYC expression and caused decreased tumor cell proliferation, apoptosis, and cell cycle arrest in Daudi cells. (**A**) RT-qPCR and WB of MYC expression upon 4 μM 17-AAG treatment over the course of four days; Tubulin is used as a WB loading control. (**B**) RT-qPCR of canonical MYC target genes: *CDK4*, *NCL*, and *ODC1* upon three days treatment of 4 μM 17-AAG or methanol control. (**C**) Growth curve of cells treated with methanol control or 4 μM 17-AAG over the course of four days. (**D**) Flow cytometry profile of AnnexinV staining (X axis) and PI (Y axis) upon three days treatment with 4 μM 17-AAG. The lower right quadrant indicates the percentage of early apoptotic cells in each condition; the upper right quadrant indicates the percentage of late apoptotic cells; the upper left quadrant indicates percentage of necrotic cells; and the left lower quadrant indicates percentage of live/non-apoptotic cells. Apoptotic cells (Annexin V-positive cells) are displayed as the percentage of gated cells. (**E**) Flow cytometric cell cycle analysis

using propidium iodide (PI) staining upon three days treatment with 4μM 17-AAG. Cell cycle distribution (G1, S and G2/M) are displayed in percent. (**F**) RT-qPCR of *CDKN2b* and *CDKN1a* upon three day treatment of 4 μM 17-AAG or methanol control. RT-qPCR was normalized to *RPL13a;* Tubulin was used as a loading control for WB. Error bars represent mean ± SEM; n = 3; two-tailed Student's *t*-test: NS = not significant; * $p < 0.05$; ** $p < 0.01$; *** $p < 0.001$.

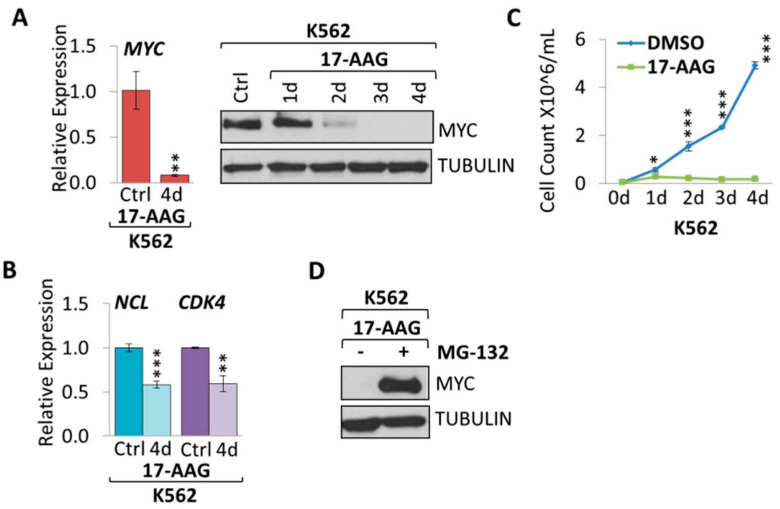

Figure S3. 17-AAG treatment causes MYC downregulation and proteasomal degradation of MYC protein in Chronic Myeloid Leukemia. (**A**) RT-qPCR and WB of MYC expression upon 4 μM 17-AAG treatment or DMSO control over the course of four days. (**B**) RT-qPCR of canonical MYC target genes: *CDK4* and *NCL*, upon four days treatment of 4 μM 17-AAG or DMSO control. (**C**) Growth curve of cells treated with DMSO control or 4 μM 17-AAG over the course of four days. (**D**) WB of MYC expression upon 4 μM 17-AAG and 10 μM MG-132 combination treatment over four hours. RT-qPCR was normalized to *RPL13a;* Tubulin was used as a loading control for WB. Error bars represent mean ± SEM; n = 3; two-tailed Student's *t*-test: * $p < 0.05$; ** $p < 0.01$; *** $p < 0.001$.

Table S1. RT-qPCR primers

Gene	Forward	Reverse
Hs *MYC*	CTGCGACGAGGAGGAGAA	GGCAGCAGCTCGAATTTCTT
Hs *CDK4*	ATGGCTACCTCTCGATATGAGC	CATTGGGGACTCTCACACTCT
Hs *NCL*	GCACCTGGAAAACGAAAGAAGG	GAAAGCCGTAGTCGGTTCTGT
Hs *ODC1*	TTTACTGCCAAGGACATTCTGG	GGAGAGCTTTTAACCACCTCAG
Hs *CDKN1A*	TGTCCGTCAGAACCCATGC	AAAGTCGAAGTTCCATCGCTC
Hs *CDKN2B*	GATGTGCAAGCGACGACAGA	GAGCAAAGGCCAGCATCCT
Hs *RPL13A*	CTGGAAGATGGTCGTACCCTG	GGTCTTGCCAGTGAGTGTCT

Table S2. ChIP-qPCR tiling primers

Amplicon	Relative/Absolute Location	Forward	Reverse
1	Chr14. 105,862,121/ 105,862,226	AGGTCACCGCGAGAGTCTAT	GCACTTTCAGATCTGGGCCT
2	Chr14. 105,582,220/ 105,582,343 and 105,701,268/ 105,701,391	GCCAGGTCTCGACTTAGCAC	TGGGGTACAAGAGGCTTCAG
3	Chr14. 105,575,299/ 105,575,593 and 105,696,320/ 105,696,508	TCCAGAAATAGCTTGCACGA	AGTAACCCAAGTGGGCCTGT
4	Chr14. 105,566,448/ 105,566,625 and 105,686,292/ 105,686,469	GGCTGGACACACTAGCAGGT	CTGCTCTGTGGTTCCCATTT
5	-2,163bp/ -2,255bp from *MYC* TSS	AACACTTGAACGCTGAGCTG	CCACCACCTCCAAAAGAGAA
6	-688bp/ -785bp from *MYC* TSS	GAGCAGCAGAGAAAGGGAGA	CAGCCGAGCACTCTAGCTCT
7	-438bp/ -534bp from *MYC* TSS	TTTATAATGCGAGGGTCTGGA	AGAAGCCCTGCCCTTCTC
8	-240bp/ -387bp from *MYC* TSS	GGGATCGCGCTGAGTATAAA	CCTATTCGCTCCGGATCTC
9	754bp/ 920bp from MYC TSS	GGGATCGCGCTGAGTATAAA	CCTATTCGCTCCGGATCTC
10	1,820bp/ 1,917bp from *MYC* TSS	AGGTGTTAGGACGTGGTGTTG	CCCTGGTTTTTCCAAGTCAA
11	2,881bp/ 2,996bp from *MYC* TSS	CGGTGCAGCCGTATTTCTAC	CAGCAGCTCGAATTTCTTCC
12	3,576bp/ 3,670bp from *MYC* TSS	AGCGACTCTGGTAAGCGAAG	AGTGGCCCGTTAAATAAGCTG

13	4,990bp/ 5,083bp from *MYC* TSS	CGATGTTGTTTCTGTGGAAAAG	GCTGTGAGGAGGTTTGCTGT
14	5,425bp/ 5,522bp from *MYC* TSS	AGCAGAGGAGCAAAAGCTCA	ACGCACAAGAGTTCCGTAGC
